# Bridging medicine and education for global developmental delay: a cluster sampling study on screening, diagnosis, and intervention in preschool children

**DOI:** 10.3389/fpubh.2026.1860897

**Published:** 2026-08-12

**Authors:** Yiyi Zhang, Lan Zhang

**Affiliations:** Chengdu Women's and Children's Central Hospital, School of Medicine, University of Electronic Science and Technology of China, Chengdu, China

**Keywords:** developmental quotient, early intervention, global developmental delay, medical-educational integration, preschool children, screening and diagnosis

## Abstract

**Background:**

Global developmental delay (GDD) is a common neurodevelopmental disorder in preschool children. Early screening and integrated intervention are critical for optimal neurodevelopment, yet robust evidence remains limited.

**Methods:**

A non-randomized controlled trial was conducted included 3,216 children aged 3–6 years for three-level GDD screening. Thirty children with confirmed GDD [defined as total General Quotient <70 on the Chinese version of the Griffiths Mental Development Scales (GDS-C)] were allocated to receive 6-month medical-educational integrated intervention or conventional training (exploratory pilot trial); both the overall General Quotient and domain-specific subscale developmental quotients (DQ) were evaluated via GDS-C.

**Results:**

The prevalence of GDD was 1.3%, with higher rates in boys and suburban areas. The integrated intervention group showed significantly greater DQ improvements in social, language, eye-hand coordination, visual performance, and practical reasoning domains (all *p* < 0.05), while no significant difference was observed in gross motor domain.

**Conclusion:**

A comprehensive multi-component intervention program, encompassing integrated medical-educational components, structured parent training, and home-based intervention measures, yielded preliminary short-term developmental benefits compared with single institutional rehabilitation.

## 1 Introduction

Global developmental delay (GDD) is one of the most prevalent neurodevelopmental disorders among children under 5 years of age, with an estimated global prevalence of 1–3% (1–2). According to the diagnostic criteria outlined in the Diagnostic and Statistical Manual of Mental Disorders, Fifth Edition (DSM-5), GDD is characterized by significant and pervasive delays across two or more core developmental domains—including gross and fine motor skills, expressive and receptive language, cognitive functioning, and personal–social abilities (3). In recent years, although advances in perinatal medicine have substantially improved the survival rates of high-risk neonates, these children concurrently face an elevated risk of subsequent neurodevelopmental impairments, thereby positioning GDD as a critical target for early health intervention during a period of heightened neural plasticity (4–5).

It is important to note that, in clinical practice, GDD is typically regarded as a provisional diagnostic label applied to children under 5 years of age who are not yet able to complete standardized intellectual assessments (3). Should developmental delays persist into school age, these children may be reclassified as having intellectual disability (ID), reflecting a clear continuity in their developmental trajectory (6–7). Longitudinal studies have demonstrated that children initially diagnosed with GDD in early childhood frequently exhibit persistent functional impairments during the school-age years, particularly in domains such as motor coordination, cognitive executive functioning, and behavioral regulation (7). These findings underscore the critical urgency of implementing effective interventions during early sensitive periods characterized by heightened neuroplasticity. Dong et al. (8) conducted a multicenter study in China involving 306 children diagnosed with GDD. Their findings demonstrated that participation in a parent-implemented early intervention program (PIEIP) led to statistically significant improvements in developmental quotients (DQ) across motor, social, and language domains (*p* < 0.05), alongside a substantial reduction in parental caregiving stress (*p* < 0.001). Multiple systematic reviews further corroborate the efficacy of early, multidisciplinary, family-centered comprehensive interventions in improving long-term outcomes for children with GDD (9–10). Nevertheless, the global landscape of GDD prevention and management remains characterized by systemic fragmentation. At the screening stage, the majority of existing tools were developed in high-income countries, and their psychometric validity—particularly reliability and cultural appropriateness—has been inadequately evaluated in low- and middle-income countries (LMICs) (11). In the diagnostic phase, the clinical uptake of recommended genetic testing remains substantially below guideline standards (10), with socioeconomic status exerting a pronounced influence on access to such services. Regarding intervention, more than 53 million children under 5 years of age worldwide require effective developmental support, yet LMICs face formidable barriers in service delivery, including shortages of trained personnel, limited infrastructure, and insufficient policy integration (12). The World Health Organization has proposed promoting the systematic integration of health and education services for children with developmental delays and disabilities through its Nurturing Care Framework (13), while Patton et al. (14), writing in The Lancet, have emphasized the urgency of bridging the gap between research, policy, and practice to build a cohesive support system that covers the whole life course of children with neurodevelopmental disorders. The integrated medical-educational model—by synergizing clinical expertise in assessment and diagnosis with educational strengths in sustained, context-sensitive intervention—represents a promising strategy to bridge these systemic gaps. This model leverages the precision of medical evaluation and the continuity of educational support, while actively engaging parents to extend and reinforce therapeutic strategies within the home environment (8, 10). Schuh et al. (15) developed Preschool and Me (PreM), an educational-medical linkage model designed to increase access to school-based services for preschool children with developmental delays and disabilities. This community-clinical linkage intervention combines personalized medical-educational care plans with remote navigator support, targeting multiple levels of influence to improve access to therapeutic and educational services. However, this model currently lacks standardized operational protocols and robust, large-scale validation across diverse sociocultural and economic contexts globally.

The epidemiology and long-term impacts of global developmental delay (GDD) represent critical priorities for scientific research and public health policy. Against this backdrop, the present study is designed with three primary objectives:

- to delineate the epidemiological characteristics of GDD among preschool-aged children and to identify its associated risk and protective factors, thereby establishing robust baseline data to inform global and regional assessments of GDD burden (16–18);
- to rigorously evaluate the effectiveness of an integrated medical-educational model across the entire continuum of GDD care—including screening, diagnosis, and intervention—in direct response to the urgent international call to bridge the persistent fragmentation between these critical service components (19);
- to develop and validate a culturally adaptive and contextually feasible framework for GDD prevention and management that can be effectively implemented at the primary healthcare level, offering scalable, evidence-based strategies and a practical reference model for low- and middle-income countries and regions worldwide. This research agenda directly addresses several key gaps in the current global landscape of child developmental health.

## 2 Materials and methods

### 2.1 Study design

This work employed multi-site cluster sampling and a three-tier screening-diagnostic pipeline to identify preschoolers with developmental delays, followed by a controlled pilot trial. Screening was conducted across nine Chengdu kindergartens covering four urban and four suburban districts from September 2023 to December 2024. Eligible children aged 36–83 months with ≥ 3 months of preschool attendance were included; children with severe sensory impairments, confirmed genetic/metabolic disorders or severe neurological diseases were excluded. Diagnosed children were assigned to an integrated medical-educational intervention or conventional rehabilitation per guardian voluntary choice.

**Figure 1:**
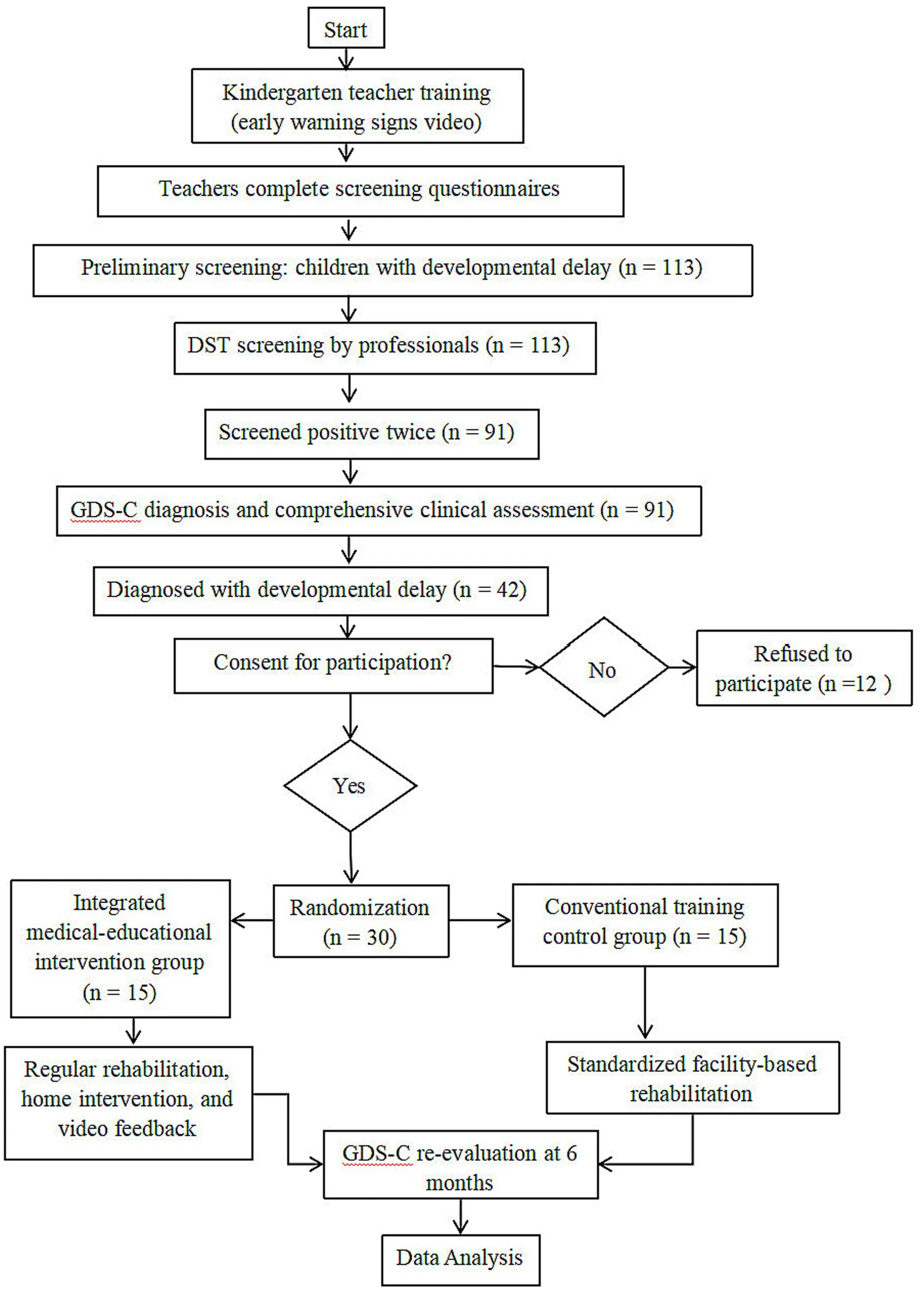
Flowchart of participant recruitment, screening, diagnosis, and procedures for the study.

The protocol was approved by the Ethics Committee of Chengdu Women’s and Children’s Central Hospital (Approval No. [2021]84). Written informed consent was collected from all guardians, and all procedures complied with the Declaration of Helsinki. This pilot trial had no prospective clinical trial registration, and formal registration will be completed for subsequent large-scale trials. The full research workflow is presented in Figure 1.

Sample size for population screening was calculated via the formula = μ2a*π*(1-*π*)/*σ*2, where μ2a = 1.962, *π* = 7.46‰, *σ* = 0.01.

Based on an assumed 8% overall developmental delay prevalence and 3% GDD prevalence, the minimum required sample size was 2,827. A total of 3,216 children were finally enrolled, meeting the power requirement for screening. For the 6-month intervention trial, 21 participants per group were pre-specified to achieve 80% statistical power (*d* = 0.8, *α* = 0.05), yet only 15 children were allocated to each arm, leading to an actual power of 0.65. Insufficient recruitment stemmed from low guardian consent rates and pre-intervention attrition, so all intervention outcomes are interpreted as preliminary exploratory results. The screened cohort consisted of 51.0% boys and 49.0% girls, with a mean age of 54.53 ± 8.95 months. Blinding was only applied to GDS-C outcome assessors; preschool teachers, caregivers and intervention therapists could not be blinded owing to obvious differences between the two intervention packages.

#### 2.1.1 Level 1 screening (warning signs screening)

The first-line screening was performed using the Warning Signs of Children’s Psychological and Behavioral Developmental Problems, a culturally adapted screening instrument developed by the Chinese Center for Disease Control and Prevention (20). This tool is designed for children aged 0–6 years and comprises 16 items distributed across four age groups (four items per age group), covering gross motor, fine motor, language, and social development domains. To ensure screening accuracy and inter-rater reliability, 36 kindergarten teachers received standardized training on instrument administration and developmental milestone recognition, and only teachers who passed the qualification assessment were authorized to conduct the screening (21).

#### 2.1.2 Level 2 screening (DST evaluation)

Children who screened positive at Level 1 underwent second-line screening using the Developmental Screening Test for Children Aged 0–6 Years (DST), a standardized instrument developed by the Pediatric Hospital of Fudan University (22). The DST was administered by five professional assessors who had completed standardized training and achieved inter-rater reliability coefficients exceeding 0.90. This tool covers three domains: motor, social adaptation, and intelligence (23). A developmental quotient (DQ) < 70 was defined as abnormal, consistent with established diagnostic criteria for developmental delay (22).

#### 2.1.3 Level 3 diagnosis (GDS-C evaluation)

Diagnostic assessment was conducted using the Chinese version of the Griffiths Mental Development Scales (GDS-C), a culturally adapted and validated instrument for assessing neurodevelopmental profiles in Chinese children aged 0–8 years (22). The GDS-C comprises six domains: locomotor (A), personal-social (B), hearing-language (C), eye-hand coordination (D), performance (E), and practical reasoning (F) (24). The scale generates two types of quotient indicators: an overall General Quotient (GQ) representing comprehensive neurodevelopmental level and domain-specific subscale Developmental Quotients (sub-DQ) reflecting performance in each developmental dimension. A total General Quotient (GQ) < 70 served as the quantitative cutoff to screen suspected GDD cases, while individual subscale DQ values were only used to characterize domain-specific developmental deficits and could not independently confirm a GDD diagnosis (2). After completing GDS-C scoring, all children with DQ < 70 received a standardized comprehensive clinical assessment conducted by two attending pediatricians with developmental-behavioral pediatric certification. The clinical evaluation covered perinatal history, developmental milestone history, behavioral observation, physical and neurological examination, family developmental history, and exclusion criteria confirmation (severe hearing/visual impairment, confirmed genetic/metabolic diseases, other neurodevelopmental disorders). The final GDD diagnostic conclusion was jointly determined by combining GDS-C quantitative results and comprehensive clinical medical evaluation, strictly following DSM-5 diagnostic criteria for Global Developmental Delay (2).

#### 2.1.4 Intervention group (comprehensive medical-educational integration package)

Building upon routine rehabilitation, the intervention group received a multicomponent medical-educational integrated intervention consisting of: (i) professional training for kindergarten teachers (40 h, covering GDD knowledge, developmental assessment, and intervention strategies); (ii) structured parent training (seven weekly sessions on home environment and play-based development); (iii) home intervention (≥ 1 h/day of structured activities with weekly video submission for expert review); and (iv) institutional rehabilitation (three sessions/week targeting motor, language, and cognitive domains).the emphasis on integrating nurturing care with health, nutrition, and early learning aligns with the Lancet Early Childhood Development Series’ framework for scalable, sustainable interventions in resource-limited settings (25).

#### 2.1.5 Control group (conventional training)

The control group received standard community-based rehabilitation (three 45-min sessions weekly, targeting motor and speech functions) without supplemental parent training or home intervention. Following a 6-month intervention period, all participants were reassessed using the GDS-C. Primary outcomes were domain-specific and overall DQ changes from baseline.

### 2.2 Statistical analysis

Statistical analyses were performed using SPSS version 25.0 (IBM Corp., Armonk, NY, USA) and R version 4.1.0 (R Foundation for Statistical Computing, Vienna, Austria). Baseline characteristics were summarized as mean ± standard deviation (SD) for continuous variables and as frequencies with percentages for categorical variables. Normality of distribution was assessed using the Shapiro–Wilk test. Between-group differences at baseline were evaluated using independent-samples *t*-tests or Mann–Whitney *U* tests for continuous variables, and chi-squared tests or Fisher’s exact tests for categorical variables, as appropriate.

Changes in developmental quotient (DQ) across domains from baseline to post-intervention were analyzed using independent-samples *t*-tests to compare between-group differences in DQ increments. To identify key factors associated with intervention efficacy while accounting for repeated measurements, Generalized estimating equations (GEE) with an exchangeable working correlation structure were applied to account for repeated developmental measurements within individual children. Post-intervention total and domain-specific DQ scores were set as dependent variables. Fixed covariates included baseline DQ, chronological age, sex, residential suburban/urban location, and the degree of parental participation, while group assignment (integrated intervention vs. conventional rehabilitation) served as the primary independent predictor.

To control for multiple comparisons across the six developmental domains, the Benjamini–Hochberg false discovery rate (FDR) procedure was applied to the six primary outcomes (domain-specific DQ changes). FDR-adjusted *p*-values are reported in Table 1 and text; adjusted *p*-values < 0.05 were considered statistically significant. The number of comparisons included in the FDR correction was six. All *p*-values presented in the main text and Table 1 are adjusted via the Benjamini–Hochberg FDR correction for six independent developmental domain comparisons; no other statistical tests in this study underwent multiple comparison adjustment.

**Table 1:** Two groups before and after intervention (*x̄* ± *s*).

Domain	Intervention group (*n* = 15)	Control group (*n* = 15)	*t*-value	*p*-values	Effect size	95% CI
Gross motor skills	14.88 ± 6.12	13.43 ± 5.84	0.542	0.592	0.12	(−0.48, 0.96)
Personal society	18.42 ± 8.65	9.70 ± 4.17	2.106	0.023	0.68	(0.50, 2.07)
Auditory language	18.85 ± 10.28	8.03 ± 5.63	2.241	0.007	0.73	(0.52, 2.09)
Hand-eye coordination	14.49 ± 5.93	9.63 ± 3.86	2.378	0.039	0.62	(0.21, 1.73)
Visual representation	9.05 ± 6.95	4.19 ± 8.02	2.463	0.017	0.58	(0.09, 1.38)
Practical reasoning	18.65 ± 7.86	10.21 ± 4.72	2.019	0.021	0.65	(0.51, 2.09)

Domain	Boys (*n* = 16)	Girls (*n* = 14)	*t*-value	*p*-values	Statistical significance	Effect size	95% CI
Gross motor skills	14.88 ± 6.12	13.43 ± 5.84	0.542	0.592	*p* > 0.05	0.24	(−0.48, 0.96)
Personal society	18.42 ± 8.65	9.70 ± 4.17	2.106	0.023	*p* < 0.05	1.26	(0.47, 2.04)
Auditory language	18.85 ± 10.28	8.03 ± 5.63	2.241	0.007	*p* < 0.01	1.28	(0.49, 2.07)
Hand-eye coordination	14.49 ± 5.93	9.63 ± 3.86	2.378	0.039	*p* < 0.05	0.96	(0.20, 1.71)
Visual representation	4.19 ± 8.02	9.05 ± 6.95	−1.780	0.097	*p* > 0.05	−0.64	(−1.38, 0.09)
Practical reasoning	18.65 ± 7.86	10.21 ± 4.72	2.019	0.021	*p* < 0.05	1.28	(0.49, 2.07)
Overall developmental quotient	4.93 ± 5.71	1.73 ± 5.29	2.184	0.025	*p* < 0.05	0.58	(−0.15, 1.31)

## 3 Results

### 3.1 Screening results and prevalence of GDD

Of 3,216 preschoolers aged 3–6 years recruited from nine Chengdu kindergartens, 113 (3.5%) screened positive at the first stage. After secondary DST screening, 42 children (1.3% of the total sample) received a definitive GDD diagnosis via GDS-C assessment (Table 2). GDD prevalence was significantly elevated among boys (1.8%) and suburban residents (1.7%) relative to girls (0.8%) and urban peers (0.8%) (both *p* < 0.05). Thirty children (15 per group) whose guardians provided written informed consent completed the 6-month intervention and constituted the final analytic sample (Figure 1).

**Table 2:** Results of GDD screening at each stage.

Screening phase	Initial screening population	Positive cases	Positive rate (%)	95%CI
Preliminary screening	3,216	113	3.5	2.9–4.2
Re-screening	113	91	2.9	2.3–3.5
Diagnosis	91	42	1.3	0.9–1.7

Our 3.5% first-stage positive rate was markedly lower than the 10.8–26.2% range documented in the scale’s normative validation study (20). This disparity stems from three critical distinctions: the validation cohort encompassed high-risk infants aged 0–3 years, whereas our sample exclusively comprised stable 3–6-year-old kindergarteners; the reference relied on hospital-based sampling prone to referral bias, while we adopted population-level cluster sampling of community preschools; and standardized training and mandatory certification for all our screeners effectively minimized false-positive screening results.

### 3.2 Baseline characteristics

Baseline characteristics were well balanced between the medical-educational integrated intervention group and the conventional training control group. No statistically significant differences were observed in sex, age, gestational age, mode of delivery, or maternal pregnancy risk factors (all *p* or *χ*^2^ > 0.05; Table 3).

**Table 3:** Comparison of general characteristics between the integrated medical-educational intervention group and the conventional training control group.

Indicator	Category	Conventional training control group (*n* = 15)	Integrated medical-educational intervention group (*n* = 15)	Statistic	*p*-value
Gender, *n* (%) (95% CI)	Boys	10 (66.67%) (42.81, 90.52%)	11 (73.33%) (50.95, 95.71%)	*χ*^2^ = 0.159	0.690
	Girls	5 (33.33%) (9.48, 57.19%)	4 (26.67%) (4.29, 49.05%)		
Age (month), mean (95% CI)	—	57.73 (52.68, 62.78)	51.33 (46.41, 56.25)	*t* = 1.947	0.062
Mode of delivery, *n* (%) (95% CI)	Cesarean section	5 (33.33%) (9.48, 57.19%)	5 (33.33%) (9.48, 57.19%)	*χ*^2^ = 0.000	1.000
	Vaginal delivery	10 (66.67%) (42.81, 90.52%)	10 (66.67%) (42.81, 90.52%)		
Gestational age (week), mean (95% CI)	—	38.25 (37.02, 39.48)	39.67 (39.35, 39.99)	*t* = −1.056	0.339
Maternal risk factors during pregnancy, *n* (%) (95% CI)	Preterm birth	2 (13.33%) (0.00, 30.54%)	4 (26.67%) (4.29, 49.05%)	*χ*^2^ = 1.733	0.785
	Hypertension	2 (13.33%) (0.00, 30.54%)	1 (6.67%) (0.00, 19.29%)		
	Hypothyroidism	2 (13.33%) (0.00, 30.54%)	1 (6.67%) (0.00, 19.29%)		

*χ*^2^ = Pearson’s chi-square test; *t* = Independent samples *t*-test. All tests were two-tailed, and a *p*-value < 0.05 was considered statistically significant.

### 3.3 Changes in developmental quotient

Following 6 months of intervention, both groups demonstrated significant within-group improvements in all domain-specific subscale developmental quotient (DQ) scores and the overall General Quotient (all *p* < 0.05). The intervention group exhibited significantly greater gains compared with the control group in the personal-social (18.42 ± 8.65 vs. 9.70 ± 4.17; *p* = 0.023, Cohen’s *d* = 0.68), hearing-language (18.85 ± 10.28 vs. 8.03 ± 5.63; *p* = 0.007, *d* = 0.73), eye-hand coordination (14.49 ± 5.93 vs. 9.63 ± 3.86; *p* = 0.039, *d* = 0.62), visual performance (9.05 ± 6.95 vs. 4.19 ± 8.02; *p* = 0.017, *d* = 0.58), and practical reasoning (18.65 ± 7.86 vs. 10.21 ± 4.72; *p* = 0.021, *d* = 0.65) domains. The intervention group also showed a numerically higher improvement in overall DQ (4.93 ± 5.71 vs. 1.73 ± 5.29; *p* = 0.025, *d* = 0.59). No significant between-group difference was observed in the gross motor domain (14.88 ± 6.12 vs. 13.43 ± 5.84; *p* = 0.592, *d* = 0.12). Detailed results are presented in Table 1.

### 3.4 Exploratory subgroup analyses and factors affecting intervention efficacy

Exploratory subgroup analyses (limited by small sample sizes: boys = 16, girls = 14; suburban = 18, urban = 12) revealed larger DQ gains in boys across personal-social, hearing-language, eye-hand coordination and practical reasoning domains (all *p* < 0.05, Cohen’s *d* = 0.96–1.28, Table 4), with no gender disparities in gross motor or visual performance. Suburban children exhibited greater improvements in personal-social, auditory language, practical reasoning and global DQ (all *p* < 0.05, Cohen’s *d* = 0.77–1.16, Table 5); trends toward superior gross motor gains among suburban children were inconclusive due to broad confidence intervals, while hand-eye coordination and visual performance did not differ by residential setting.

**Table 4:** Comparison of DQ increments across developmental domains by gender (mean ± SD, months).

Domain	Boys (*n* = 16)	Girl (*n* = 14)	*t*-value	*p*-values	Statistical significance
Gross motor skills	14.88 ± 6.12	13.43 ± 5.84	0.542	0.592	*p* > 0.05
Personal society	18.42 ± 8.65	9.70 ± 4.17	2.106	0.023	*p* < 0.05
Auditory language	18.85 ± 10.28	8.03 ± 5.63	2.241	0.007	*p* < 0.01
Hand-eye coordination	14.49 ± 5.93	9.63 ± 3.86	2.378	0.039	*p* < 0.05
Visual representation	4.19 ± 8.02	9.05 ± 6.95	−1.780	0.097	*p* > 0.05
Practical reasoning	18.65 ± 7.86	10.21 ± 4.72	2.019	0.021	*p* < 0.05
Overall developmental quotient	4.93 ± 5.71	1.73 ± 5.29	2.184	0.025	*p* < 0.05

**Table 5:** Comparison of DQ increments across developmental domains in children from different regions (mean ± SD, months).

Domain	Suburban group (*n* = 18)	Urban core group (*n* = 12)	*t*-value	*p*-values	Statistical significance	Effect size	95% CI
Gross motor skills	10.2 ± 2.9	8.7 ± 2.5	2.01	0.045	*p* < 0.05	0.58	(−0.20, 1.29)
Personal society	13.6 ± 3.4	9.8 ± 3.1	2.28	0.031	*p* < 0.05	0.65	(0.37, 1.94)
Auditory language	11.3 ± 3.2	8.1 ± 2.7	2.15	0.041	*p* < 0.05	0.61	(0.28, 1.84)
Hand-eye coordination	12.7 ± 3.1	10.3 ± 2.9	1.89	0.067	*p* > 0.05	0.54	(0.04, 1.55)
Visual representation	9.8 ± 2.7	8.9 ± 2.4	1.45	0.156	*p* > 0.05	0.42	(−0.39, 1.08)
Practical reasoning	11.9 ± 3.3	9.5 ± 2.8	2.07	0.047	*p* < 0.05	0.59	(0.01, 1.53)
Overall developmental quotient	11.4 ± 2.8	9.2 ± 2.6	2.24	0.033	*p* < 0.05	0.64	(0.05, 1.57)

GEE models confirmed integrated intervention, baseline developmental status and parental engagement as independent positive predictors of developmental gains, with sex and residence as significant effect modifiers (all *p* < 0.05, Table 6). Prospective compliance data validated the mediating role of caregiver participation. Across 6 months, the intervention group achieved a 92.7% average weekly home-intervention video submission rate (96.1% suburban vs. 87.4% urban) and 91.1% aggregate attendance at parent training sessions (97.2% suburban vs. 82.8% urban). Higher adherence among suburban families likely contributed to their more pronounced developmental improvements.

**Table 6:** GEE analysis: key factors influencing intervention efficacy.

Influencing factors	Wald *χ*^2^	*p*值	Exp(B)	95%CI
Intervention approach (integration of medical and educational services)	8.92	0.003	2.36	(1.35–4.12)
Baseline developmental level	6.78	0.009	1.89	(1.17–3.05)
Parental involvement	7.35	0.007	2.14	(1.23–3.72)
Gender (male)	4.26	0.039	1.62	(1.02–2.56)
In suburban areas	4.89	0.027	1.75	(1.07–2.87)

## 4 Discussion

### 4.1 Epidemiological characteristics and mechanisms of intervention efficacy

The prevalence of GDD observed in this study (1.3%) is consistent with global estimates, which range from 1 to 3% in children under 5 years (26–27). This prevalence is lower than that reported in some developing countries, a discrepancy that may reflect regional variations in economic development, healthcare infrastructure, and screening methodologies (17). We found a significantly higher incidence of GDD among boys than girls, aligning with the well-established male predominance in neurodevelopmental disorders (28–30). From a neurobiological perspective, it is hypothesized that fluctuations in fetal and neonatal testosterone levels occurring during critical windows of brain sexual differentiation might correlate with variations in cerebral lateralization, which could partially explain the higher vulnerability to neurodevelopmental differences observed in male children. However, such biological pathways remain unconfirmed within the current small sample and require targeted mechanistic research for validation (31–32). From a socio-environmental perspective, disparities in family rearing styles and divergent societal developmental expectations for boys represent one plausible contextual factor that may be associated with the gender-based gaps in developmental progress detected in this exploratory cohort (33).

The prevalence of GDD among suburban children was approximately 2.13-fold higher than that recorded for children residing in central urban districts. Several contextual factors are proposed as potential correlative explanations for this observed disparity, though none can be confirmed as direct causal drivers given the limitations of our exploratory sampling design. Firstly, suburban communities generally face relative shortages of systematic early childhood educational resources, which may reduce consistent developmental stimulation opportunities for young children (34). Secondly, caregivers living in suburban settings might possess less awareness of typical developmental milestones and early warning signs of delay, which could potentially lead to delayed formal screening and diagnosis (35). Thirdly, suburban regions commonly have more restricted access to specialized pediatric developmental clinics and standardized intervention services compared to urban core areas, a contextual feature that may co-occur with elevated GDD detection rates in this sample (36). Future large-scale controlled studies are needed to disentangle the independent contributions of these contextual variables. These contextual factors suggest that GDD prevention and control efforts should pay special attention to rural and remote regions.

The age-specific distribution of warning signs provides important guidance for targeted intervention. The study revealed that the primary problems in children aged 3–4 years were concentrated in the fine motor domain, whereas children aged 5–6 years more frequently exhibited motor coordination and social difficulties. This pattern aligns with typical neurodevelopmental trajectories and underscores the need for age-specific screening and intervention strategies (37–38).

A finding is that the advantage of the medical-educational integrated model exhibits group specificity. This model showed particularly pronounced benefits for boys in higher-order functional domains such as social interaction and language (39). While exploratory subgroup analyses suggested differential effects by sex and region, these findings are hypothesis-generating only due to very small subgroup sizes (boys *n* = 16, girls *n* = 14; suburban *n* = 18, urban *n* = 12). Any biological or sociological interpretations (e.g., testosterone effects, resource substitution) are speculative and require verification in adequately powered studies (40–41).

Finally, we reaffirm that parental involvement serves as an intrinsic pathway in intervening for children with global developmental delay. The high effect size of parental engagement (Exp(B) = 2.14, Table 6) is intrinsically linked to the inter-group and regional outcome differences shown in Tables 1, 4, which is further supported by objective compliance statistics collected during the trial. Suburban families, possibly due to lifestyle rhythms and stronger educational anxiety regarding their children’s developmental progress, exhibited higher video submission and parent training attendance rates than urban families, reflecting greater compliance and sustained investment in daily home-based intervention plans that ultimately amplified developmental intervention gains (8). This empirical evidence confirms that parental involvement acts as a critical mediating pathway through which integrated intervention programs exert their long-term developmental effects (42).

### 4.2 Discussion on the mechanisms of the medical-educational integration model

The comprehensive multi-component intervention combining medical assessment, preschool support, and home training produced greater developmental improvements in most domains; we cannot fully disentangle whether these gains originate from cross-sector integration, increased training frequency, or intensive parental involvement. Nonetheless, combined with the present and previous research evidence, this model is recognized to have the following potential mechanistic benefits.

#### 4.2.1 The integrative effect of multidisciplinary collaboration

The medical-educational integration model effectively combines professional resources from medical, educational, and family domains, forming a synergistic intervention network (8). Medical institutions provide specialized assessment and diagnosis, ensuring the relevance and scientific rigor of interventions (43). Educational settings leverage their environmental advantages to offer continuous and naturalistic intervention contexts (44). Families, through daily life practice, promote the generalization and consolidation of acquired skills (12). This multidisciplinary collaborative approach overcomes the limitations of traditional single-domain interventions, providing comprehensive support for the child (45).

#### 4.2.2 The central role of family involvement

In this study, the intervention group demonstrated significantly enhanced depth and breadth of family involvement through systematic parent training and guidance. Research indicates that parent-implemented interventions not only increase the intensity and frequency of intervention but also promote children’s socio-emotional development by improving the quality of parent–child interactions (46). Furthermore, the knowledge and skills acquired by parents during the intervention process help establish a sustained supportive environment, which is crucial for the long-term maintenance of intervention effects (47).

#### 4.2.3 The advantage of learning in natural settings

The integrated medical-educational model emphasizes intervention in natural environments, a feature that may explain its superior outcomes in domains such as social and language development. In preschool and home settings, children have more opportunities to practice and apply new skills in authentic contexts; such situated learning facilitates knowledge transfer and generalization (48). In contrast, institution-based training often lacks this natural contextual support, which may result in difficulties generalizing skills to daily life.

It is noteworthy that no significant difference was observed between the two groups in the domain of gross motor skills, which may be related to the developmental characteristics of motor abilities. Gross motor development relies more heavily on physiological maturation and repetitive practice and is relatively less sensitive to educational interventions (49). This finding suggests that intervention strategies should be tailored to different developmental domains; motor training may require more targeted physiological rehabilitation measures.

### 4.3 Comparative analysis with existing research

The findings of this study align with recent international research while also revealing several distinctive observations. Compared to conventional intervention approaches, the present research optimized intervention intensity and parental involvement, leading to improved outcomes (50). A key feature of this study is the extension of the intervention setting from specialized institutions to educational environments and households, thereby enhancing ecological validity (44).

While statistically significant DQ improvements were observed, their clinical meaningfulness remains to be established. A DQ change of ≥5 points is often considered minimally clinically important; our observed total DQ difference (3.2 points) approaches but does not clearly exceed this threshold. Functional implications for daily living, school readiness, and long-term outcomes require further investigation.

The 6-month intervention period precludes assessment of long-term sustainability. Given the chronic nature of GDD, longer follow-up is essential to determine whether short-term DQ gains translate into sustained functional improvements, school readiness, and reduced disability. A 12-month follow-up is currently underway.

### 4.4 Limitations and future directions

The intervention phase comprised only 30 participants, yielding insufficient statistical power to detect modest effects; consequently, negative findings should be interpreted with caution and all results are considered preliminary. The intervention group received a multicomponent package encompassing teacher training, parent training, structured home intervention, and weekly video feedback in addition to routine rehabilitation, whereas the control group received routine rehabilitation alone. The observed effects may therefore reflect greater overall intervention intensity or enhanced parental involvement rather than medical–educational integration perse, and factorial or dismantling designs will be needed to isolate the specific contributions of individual components. Subgroup analyses were further limited by small cell sizes, rendering any sex or region-specific interpretations speculative. Outcome assessors conducting GDS-C evaluations were blinded to group allocation; however, participants and interventionists could not be blinded due to the nature of the intervention. Finally, the single-center design and relatively homogeneous sample limit generalizability to other settings and populations. The non-randomized design introduces potential selection bias, as parents who chose the integrated intervention may differ systematically from those who chose conventional training in terms of motivation, socioeconomic resources, or perceived child severity.

### 4.5 Research significance and practical value

This pilot study provides preliminary evidence that a medical-educational integrated model effectively improves developmental outcomes in children with Global Developmental Delay (GDD), offering both theoretical contributions and translational implications. Theoretically, the findings are grounded in the ecological systems perspective, which conceptualizes child development as a dynamic process emerging from reciprocal interactions across nested environmental contexts—including immediate settings, interpersonal connections, institutional structures, and broader sociocultural forces—and posits that effective intervention necessitates the synergistic alignment of resources spanning these interconnected layers. This integrated approach resonates with contemporary frameworks emphasizing that early intervention efficacy is maximized when family-centered care, interprofessional collaboration, and progress monitoring are incorporated within a unified service delivery model (51). Furthermore, the results substantiate the principle of neuroplasticity (52), demonstrating that structured, early stimulation during critical developmental windows can induce measurable improvements in brain function and developmental trajectories. Clinically, this study establishes an operational workflow for GDD screening, diagnosis, and intervention that is readily adaptable to primary child healthcare settings. Notably, by leveraging kindergarten teachers as frontline screening personnel and co-interventionists, the model effectively mitigates the shortage of specialized professionals—a strategy particularly salient for low-resource and rural settings where access to developmental specialists remains severely constrained (53). These findings support the integration of standardized GDD screening into routine child health surveillance programs (54).and underscore the imperative for strengthened intersectoral collaboration between healthcare and education systems. Given the disproportionate burden of unmet developmental needs in rural and remote areas, the study calls for targeted policy investment, workforce capacity-building, and equitable resource allocation to ensure scalable, sustainable implementation of early developmental support services (55).

## 5 Conclusion

This 6-month integrated medical-educational intervention preliminarily showed potential developmental benefits for preschool children with GDD versus standard rehabilitation. Participants in this program achieved larger DQ gains in most developmental domains except gross motor skills. Exploratory subgroup analyses suggested boys and suburban children might benefit more. GEE models found intervention type, baseline development and parental involvement correlated with better progress. These tentative results imply this combined support model could be a feasible option for children with GDD, yet small sample and unequal intervention components restrict solid causal conclusions; larger trials are needed for further verification.

## Data Availability

The original contributions presented in the study are included in the article/supplementary material, further inquiries can be directed to the corresponding author.
